# Deep Ultraviolet Excitation Photoluminescence Characteristics and Correlative Investigation of Al-Rich AlGaN Films on Sapphire

**DOI:** 10.3390/nano14211769

**Published:** 2024-11-04

**Authors:** Zhe Chuan Feng, Ming Tian, Xiong Zhang, Manika Tun Nafisa, Yao Liu, Jeffrey Yiin, Benjamin Klein, Ian Ferguson

**Affiliations:** 1 Southern Polytechnic College of Engineering and Engineering Technology, Kennesaw State University, Marietta, GA 30060, USA; mnafisa@students.kennesaw.edu (M.T.N.); jyiin@kennesaw.edu (J.Y.); bklein8@kennesaw.edu (B.K.); ianf@kennesaw.edu (I.F.); 2 Key Laboratory of MEMS of Ministry of Education, School of Integrated Circuits, Southeast University, Nanjing 210096, China; 230208647@seu.edu.cn; 3 Advanced Photonics Center, Southeast University, Nanjing 210096, China; xzhang62@aliyun.com; 4 Research Center for Optoelectronic Materials and Devices, Guangxi Key Laboratory for the Relativistic Astrophysics, School of Physical Science & Technology, Guangxi University, Nanning 530004, China; malena326@126.com

**Keywords:** AlGaN-GaN, high x(Al), metal–organic chemical vapor deposition, spectroscopic ellipsometry, X-ray diffraction, Raman scattering, photoluminescence, temperature-dependent and time-resolved photoluminescence

## Abstract

AlGaN is attractive for fabricating deep ultraviolet (DUV) optoelectronic and electronic devices of light-emitting diodes (LEDs), photodetectors, high-electron-mobility field-effect transistors (HEMTs), etc. We investigated the quality and optical properties of Al_x_Ga_1−x_N films with high Al fractions (60–87%) grown on sapphire substrates, including AlN nucleation and buffer layers, by metal–organic chemical vapor deposition (MOCVD). They were initially investigated by high-resolution X-ray diffraction (HR-XRD) and Raman scattering (RS). A set of formulas was deduced to precisely determine x(Al) from HR-XRD data. Screw dislocation densities in AlGaN and AlN layers were deduced. DUV (266 nm) excitation RS clearly exhibits AlGaN Raman features far superior to visible RS. The simulation on the AlGaN longitudinal optical (LO) phonon modes determined the carrier concentrations in the AlGaN layers. The spatial correlation model (SCM) analyses on E_2_(high) modes examined the AlGaN and AlN layer properties. These high-x(Al) Al_x_Ga_1−x_N films possess large energy gaps E_g_ in the range of 5.0–5.6 eV and are excited by a DUV 213 nm (5.8 eV) laser for room temperature (RT) photoluminescence (PL) and temperature-dependent photoluminescence (TDPL) studies. The obtained RTPL bands were deconvoluted with two Gaussian bands, indicating cross-bandgap emission, phonon replicas, and variation with x(Al). TDPL spectra at 20–300 K of Al_0.87_Ga_0.13_N exhibit the T-dependences of the band-edge luminescence near 5.6 eV and the phonon replicas. According to the Arrhenius fitting diagram of the TDPL spectra, the activation energy (19.6 meV) associated with the luminescence process is acquired. In addition, the combined PL and time-resolved photoluminescence (TRPL) spectroscopic system with DUV 213 nm pulse excitation was applied to measure a typical AlGaN multiple-quantum well (MQW). The RT TRPL decay spectra were obtained at four wavelengths and fitted by two exponentials with fast and slow decay times of ~0.2 ns and 1–2 ns, respectively. Comprehensive studies on these Al-rich AlGaN epi-films and a typical AlGaN MQW are achieved with unique and significant results, which are useful to researchers in the field.

## 1. Introduction

Wide-bandgap nitride-based semiconductors, devices, and applications have been greatly developed in recent decades [1,2,3]. Ultra-wide-gap (UWG) semiconductors (E_g_ > 5 eV), including AlN, diamond, β-Ga_2_O_3_, and AlGaN with high Al compositions, possess excellent material properties for promoting the development of the next generation of power electronics. Al_x_Ga_1−x_N materials cover the energy range between 3.4 eV (x = 0) and 6.2 eV (x = 1) and high-x (x > 50%). Al_x_Ga_1−x_N is very attractive in deep ultraviolet (DUV) devices and applications. In the past two decades, intense research and development (R&D) emerged on high-x Al_x_Ga_1−x_N epi-materials [4,5,6,7,8,9,10,11,12,13,14], Al-rich AlGaN multiple-quantum wells (MQWs) [15,16], AlGaN DUV light-emitting diodes (LEDs) [17,18], AlGaN DUV laser diodes (LDs) [19], high-x(Al) AlGaN field-effect transistors (FETs) [20,21], Al-rich high-electron-mobility transistors (HEMTs) [22,23,24,25], and so on. R&D on Al-rich AlGaN materials and devices are the current frontiers and hot points in the field [9,10,11,12,13,14,16,17,18,19,22,23,24,25].

In the current work, we investigated the optical and structural properties of Al_x_Ga_1−x_N films with high x(Al) (60%, 71%, 75%, 81%, 87%) fractions grown on C-plane sapphire substrates with a 20 nm AlN nucleation layer and an AlN buffer layer by metal–organic chemical vapor deposition (MOCVD). To characterize these AlGaN/AlN/sapphire structures well via high-resolution X-ray diffraction (HR-XRD), we deduced a set of formulas to precisely determine the x(Al) from three orders of HR-XRD data. Screw dislocation densities in AlGaN and AlN layers were deduced. The AlGaN/AlN/sapphire structures were also characterized by visible and DUV Raman scattering (RS). The DUV (266 nm) excitation RS clearly exhibited AlGaN Raman features far superior to visible RS. Two types of simulation methods were applied to analyze the Raman longitudinal optical (LO) and E_2_(high) phonon modes. The carrier concentrations in the AlGaN layers were determined via simulation on the AlGaN longitudinal optical (LO) phonon modes. The Raman line shapes of E_2_(high) modes were analyzed by the spatial correlation model (SCM), which qualitatively investigated the AlGaN and AlN layer properties. Room temperature (RT) photoluminescence (PL) and temperature-dependent photoluminescence (TDPL) measurements were carried under the excitation from a DUV 213 nm (5.8 eV) laser to investigate these high-x(Al) Al_x_Ga_1−x_N films with energy gaps E_g_ between 5.0 and 5.6 eV. The obtained PL bands were deconvoluted with Gaussian bands, indicating cross-bandgap emission, phonon replicas, and variation with x(Al). TDPL spectra at 20–300 K of Al_0.87_Ga_0.13_N exhibit the T-dependences of the band-edge luminescence near 5.6 eV and the phonon replicas. According to the Arrhenius fitting diagram of the TDPL spectra, the activation energy (19.6 meV) associated with the luminescent process is acquired. In addition, a combined PL and time-resolved photoluminescence (TRPL) spectroscopic system with DUV 213 nm pulse excitation was applied to investigate AlGaN multiple-quantum wells (MQWs). RT TRPL decay spectra were measured at four wavelengths and fitted by two exponentials, with fast and slow decay times obtained. Comprehensive findings on the material qualities and optical properties of Al-rich AlGaN epi-films and a typical AlGaN MQW are achieved with attractive results, which provide useful references to the R&D in AlGaN and related materials.

## 2. Materials and Methods

For the material growth procedure on the C-plane sapphire substrate by metal–organic chemical vapor deposition (MOCVD), a low-temperature (LT)-AlN nucleation layer of 20 nm was first grown at 600 °C. Then, an HT-AlN buffer layer was grown at an increased temperature of 1050 °C; subsequently, Al_x_Ga_1−x_N layers with different x(Al) compositions were grown at the same temperature of 1050 °C. Precursors of trimethyl-aluminum (TMAl), trimethyl-gallium (TMGa), and ammonia (NH3) were used for Al, Ga, and N, respectively. The growth details are like those reported in [26]. The experimental samples are named A60, A71, A75, A81, and A87, with x(Al) in Al_x_Ga_1−x_N of 60.2%, 71.4%, 75.3%, 81.1%, and 87.7% determined in this study, respectively. An additional sample A35 with lower x(Al) of 35.0% is used for reference as performing XRD measurements. The AlGaN layer thicknesses are in the range of 400–600 nm, determined from spectroscopic ellipsometry (SE) measurements, like those reported in [27].

In the present work, high-resolution X-ray diffraction (HR-XRD) measurements were conducted from a system of Bruker D8 Discover, Ettlingen, Germany. SE measurements were carried out by using a Mueller matrix ellipsometer, model ME-L, from Wuhan Eoptics Technology Co. Ltd., Wuhan, China, with five or three incident angles of 50–70°. DUV 266 nm excitation Raman scattering measurements were performed at room temperature (RT), by using a confocal microscope optical system, including two lasers of 266 nm and 532 nm, and a spectrometer of iHR550 (Horiba, Irvine CA, USA) with gratings of 600 g/mm and 2400 g/mm. A combined photoluminescence (PL) and time-resolved photoluminescence (TRPL) spectroscopic system with deep ultraviolet (DUV) 213 nm excitation was built up and applied to measure AlGaN multiple quantum wells (MQWs) and high Al-composition AlGaN epi-films as well as other ultrawide bandgap (UBG) materials and structures. In DUV 213 nm excitation steady-state photoluminescence (SS-PL), temperature-dependent (TD) PL, and time-resolved PL (TRPL) experiments, samples were excited by a CNI FL-213-Pico 213 nm picosecond laser. The PL decay curves were recorded by a time-correlated single-photon-counting (TCSPC) system, as in [28]. DUV 193 nm excitation PL measurements were also conducted with a 193 nm laser source, and UV–visible optical transition measurements were carried out using a UV–visible spectrophotometer (Zolix OmniAs, Beijing, China) with deuterium lamps, as described in [29].

## 3. Results and Discussions

### 3.1. High-Resolution X-Ray Diffraction Analysis

**Figure 1:**
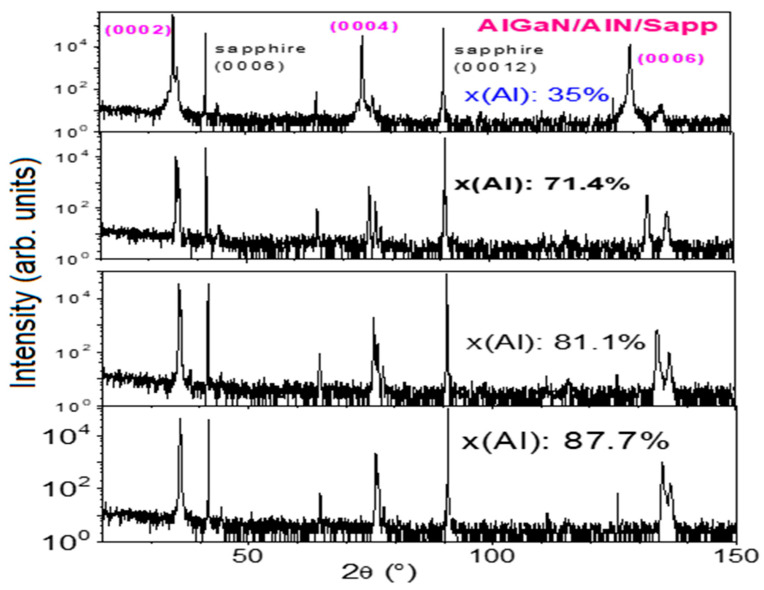
HR-XRD wide scans of four AlGaN samples, with the substrate sapphire (0006) peak at 41.70° for calibration.

Figure 1 shows high-resolution X-ray diffraction (HR-XRD) scans of four AlGaN/AlN/sapphire samples, with the substrate sapphire (0006) peaking at 41.70° for calibration.

**Figure 2:**
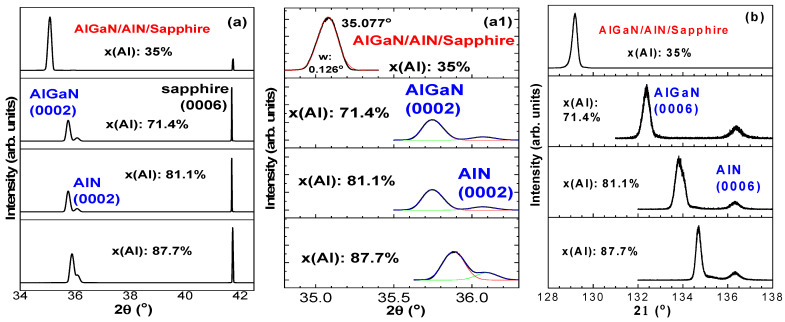
HR-XRD fine scans of four AlGaN/AlN/sapphire samples. (**a**) The first order with the sapphire (0006) peak at 41.70° for calibration, (**a1**) Gaussian fits for AlGaN (0002) peaks with red lines and for AlN (0002) peaks with green lines, and (**b**) the third-order scans.

Figure 2 exhibits the first and third fine scans of four AlGaN samples. Figure 2a presents the first-order XRD fine scan with the (0002) AlGaN and AlN peaks very close, especially for high x(Al) samples. The Gaussian fittings are made in Figure 2(a1). The fitted values for AlGaN (0002) and AlN (0002) peaks and widths are A35: 35.077, 0.126; A71: 35.749, 0.129 and 36.070, 0.157; A81: 35.749, 0.129 and 36.070, 0.157; A87: 35.884, 0.137 and 36.094, 0.141, respectively, which are used for calculations in the later part of this section.

**Figure 3:**
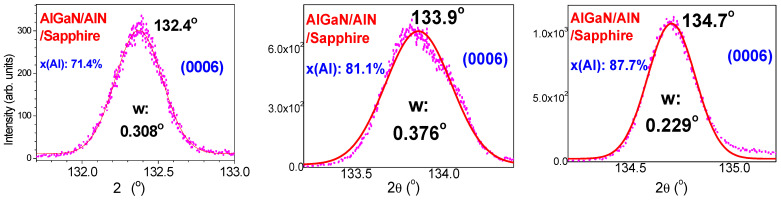
HR-XRD fine scans and Gaussian fits of the AlGaN (0006) peaks for three AlGaN samples, with high x(Al) compositions of 71.4%, 81.1%, and 87.7%, respectively.

In addition, Figure 3 exhibits HR-XRD fine scans and Gaussian fits of the AlGaN (0006) peaks for three AlGaN samples, which are confirmed with their high x(Al) composition values of 71.4%, 81.1%, and 87.7%, respectively, from calculations below.

A set of formulas can be deduced to calculate the x(Al) for experimental samples. Based upon the Bragg rule for crystals,

nλ = 2d sinθ(1)

with c = d = nλ/2sinθ, where c is the lattice constant along c-axis, λ is the X-ray wavelength of 0.154056 nm, θ the X-ray incident angle, for n = 2,

c(GaN) = λ/sinθ_GaN_, c(AlN) = λ/sinθ_AlN_, and c(AlGaN) = λ/sinθ_AlGaN_. (2)

As it is applied to the AlGaN lattice:

c(AlGaN) = c(GaN) − x[c(GaN) − c(AlN)], (3)

using c(GaN) = 0.5185 nm, c(AlN) = 0.4978 nm [30]. We can have

x = [c(GaN) − c(AlGaN)]/[c(GaN) − c(AlN)]          = [{λ/sinθ_GaN_} − {λ/sinθ_AlGaN_}]/[{λ/sinθ_GaN_} − {λ/sinθ_AlN_}]           = [{1/sinθ_GaN_} − {1/sinθ_AlGaN_}]/[{1/sinθ_GaN_} − {1/sinθ_AlN_}] (4)

From XRD values of GaN with 2θ_GaN1_(0002) = 34.60°, 2θ_GaN3_(0006) = 126.20° [31], and AlN data with 2θ_AlN1_(0002) = 36.10°, 2θ_AlN3_(0006) = 136.30° [32,33], we can deduce brief calculation expressions based upon (0002) and (0006) XRD patterns as follows:

(0002), x(Al) = 7.19 × [3.367 − (1/sinθ_AlGaN1_)]; (0006), x(Al) = 22.7 × [1.121 − (1/sinθ_AlGaN3_)] (5)

By using Equation (5) and the XRD data of (0002) and (0006) patterns, we can obtain the x(Al) values in Al_x_Ga_1−x_N samples of 35.0%, 60.2%, 71.4%, 75.3%, 81.1%, and 87.7% for A35, A60, A71, A75, A81, and A87, respectively, with error bars of about ±0.2%. These values are marked in the related graphs and mentioned in Section 2. These formulas can be useful for people working in the field, although we only apply them in a limited manner in this paper.

Next, we process how to determine dislocation densities in our AlGaN layers. Referring to [14,33], the dislocation densities of Al_x_Ga_1−x_N thin films can be determined by

D_screw_ = β^2^/(4.36b^2^), (6)

where β is the FWHM of XRD (0002) peak and b = 5.1855 Å is the Burgers vector length for the screw-type threading dislocation (TD) along the *c*-axis. We can calculate the screw dislocation densities of four AlGaN films with x(Al) of 35.0%, 71.4%, 81.1%, and 87.7%, listed in Table 1. It is obtained that three AlGaN films with x(Al) of 35%, 71.4%, and 81.1% have their screw dislocation densities of about 4 × 10^18^ cm^−3^, while the high x(Al) (87.7%) sample possesses a high dislocation density beyond 7 × 10^18^ cm^−3^.

Further, we can calculate the screw dislocation densities of AlN layers in three Al_x_Ga_1−x_N/AlN/sapphire samples with x(Al) of 71.4%, 81.1%, and 87.7%. In this case of AlN, b = 0.4982 nm [33] for calculations in Table 2.

It is obtained that three AlGaN/AlN/sapphire samples with x(Al) of 71.4%, 81.1%, and 87.7% have their AlN buffer screw dislocation densities of 6–7 × 10^18^ cm^−3^, while the high x(Al) (87.7%) sample possesses a lowest AlN dislocation density of 6 × 10^18^ cm^−3^.

### 3.2. Spectroscopic Ellipsometry Analysis

**Figure 4:**
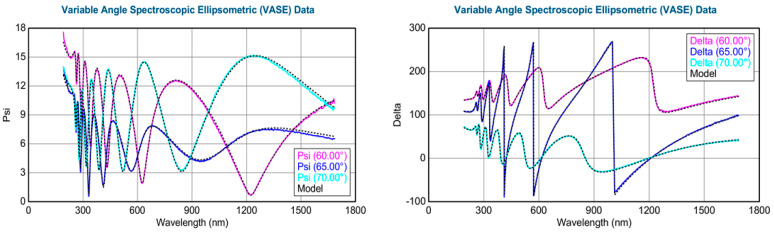
Typical SE psi (Ψ) and delta (∆) spectra at 60°–70° incidences from an Al_x_Ga_1−x_N sample A60 with x = 60.2%.

Variable angle (VA) spectroscopic ellipsometry (SE) measurements were conducted for five AlGaN-GaN HEMTs in the wavelength range of 193–1650 nm and with variable incident angles between 50 and 70 degrees, on each sample. Figure 4 shows typical VASE psi (Ψ) and delta (∆) spectra at 60°–70° incidences from an Al_x_Ga_1−x_N sample of x = 60.2%. Through the CompleteEASE software simulation (https://www.jawoollam.com/ellipsometry-software/completeease, accessed on 29 October 2024), displayed with dotted lines for all curves in the figure, the thicknesses for AlGaN layer of 470 nm and AlN buffer/nucleation layer of 70 nm were obtained, like those reported in [27]. We employed the SE technology to deduce the relationships of refraction index n and extinction coefficient k versus wavelength λ, i.e., n~λ and k~λ, like previously for other epitaxial AlGaN [27]. In the present investigation, all AlGaN/AlN/sapphire samples were performed for SE measurements and simulations with the AlGaN layer thicknesses in the range of 400–600 nm and AlN buffer/nucleation layer thickness of 70–80 nm.

### 3.3. Raman Spectroscopy Analysis

**Figure 5:**
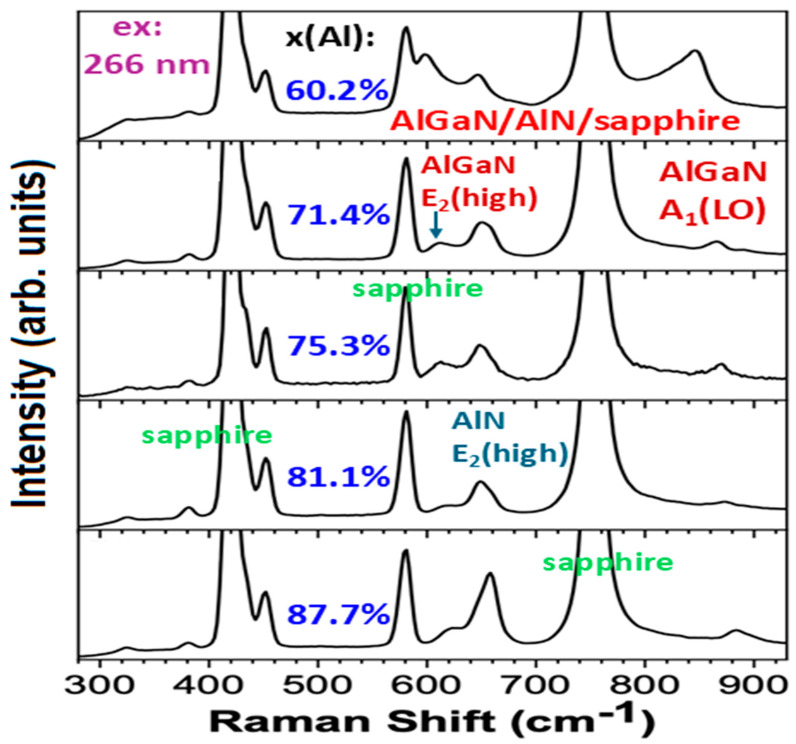
DUV 266 nm excitation Raman scattering spectra for 5 AlGaN/AlN/sapphire samples with high x(Al) between 60.2% and 87.7%, respectively.

Figure 5 shows Raman scattering spectra under the 266 nm laser excitation for five AlGaN/AlN/sapphire samples with x(Al) between 60.2% and 87.7%, respectively.

As we performed the visible 532 nm excitation Raman experiments on these samples, sapphire features at 320–460 cm^−1^, 580 cm^−1^, and 750 cm^−1^ are extremely stronger, leading to the nitride Raman features of AlGaN E_2_(high), AlN E_2_(high), and AlGaN A_1_(LO) being unrecognized or weak. These difficulties can be overcome by using DUV 266 nm excitation, as observed at Figure 5, in which Raman modes of AlGaN E_2_(high), AlN E_2_(high), and AlGaN A_1_(LO), are well recognized. In Figure 5, on the right, between 800 cm^−1^ and 930 cm^−1^, a single mode is displayed, which is the A_1_(LO) mode from five Al_x_Ga_1−x_N with the peak frequency varied on x(Al) from ~840 cm^−1^ to ~880 cm^−1^ as x(Al) increases from 60.2% to 87.7%.

Based upon theoretical analyses on Raman LO mode, the carrier concentrations can be calculated by way of the LO-phonon and plasma coupling (LOPC). To measure the free carrier concentration in wide bandgap semiconductors, a set of formulas on the Raman intensity of LOPC mode are presented as [30] follows:

(7) $$I_{LOPC}={\left.\frac{d^{2}S}{d\omega d\Omega }\right|}_{A}=\frac{16\pi hn_{2}}{V_{0}^{2}n_{1}}\frac{\omega_{2}^{4}}{C^{4}}\left(\frac{d\alpha }{dE}\right)\left(n_{\infty }+1\right)AIm\left(-\frac{1}{\varepsilon }\right)\;$$

(8) $$\begin{array}{c} \mathrm{where}\;A=1+2C\frac{\omega_{T}^{2}}{\Delta }\left[\omega_{p}^{2}\gamma \left(\omega_{T}^{2}-\omega^{2}\right)-\omega^{2}\eta \left(\omega^{2}+\gamma^{2}-\omega_{p}^{2}\right)\right]+C^{2}\left(\frac{\omega_{T}^{4}}{\Delta \left(\omega_{L}^{2}-\omega_{T}^{2}\right)}\right)\\ \;\;\;\;\;\times \left\{\omega_{p}^{2}\left[\gamma \left(\omega_{L}^{2}-\omega_{T}^{2}\right)+\eta \left(\omega_{p}^{2}-2\omega^{2}\right)\;\right]+\omega^{2}\eta \left(\omega^{2}+\gamma^{2}\right)\right\} \end{array}$$

(9) $$\Delta =\omega_{p}^{2}\gamma \left[{\left(\omega_{T}^{2}-\omega^{2}\right)}^{2}+{\left(\omega \eta \right)}^{2}\right]+\omega^{2}\eta \left(\omega_{L}^{2}-\omega_{T}^{2}\right)\left(\omega^{2}+\gamma^{2}\right)$$

In Equation (7), *n*_1_ and *n*_2_ are refractive indices at incident frequency *ω*_1_ and scattering frequency *ω*_2_, respectively; *C* is Faust–Henry coefficient, here the value is about 0.35; *α* is polarizability; *E* is macroscopic electric field; *n_ω_* is the Bose–Einstein factor. In Equations (8) and (9), *ω_p_* is the plasma frequency, $\omega_{L}$ is the longitudinal optical mode frequency; $\omega_{T}$ is transverse optical mode frequency; *η* is phonon damping constant; γ is plasma damping constant.

Followed, the dielectric function can be described as

(10) $$\varepsilon =\varepsilon_{\infty }\left(1+\frac{\omega_{L}^{2}-\omega_{T}^{2}}{\omega_{T}^{2}-\omega^{2}-i\omega \eta }-\frac{\omega_{p}^{2}}{\omega \left(\omega +i\gamma \right)}\right)$$

(11) $$\omega_{p}^{2}=\frac{4\pi ne^{2}}{\varepsilon_{\infty }m^{*}}$$

where *ω_p_* is the plasma frequency; *n* is free carrier concentration; m* is effective mass while *e* is unit charge; *ε_∞_* is high frequency dielectric constant. Equation (10) of the dielectric function has been widely used in Raman studies on various semiconductors [30]. In addition, Equation (10) was employed by D.T. Talwar et al. to investigate BeTe, Be_x_Zn_1−x_Te, p-BeTe epilayers, and BeTe/ZnTe/GaAs superlattices [34], and GeC/Si [35].

For polar semiconductors, there exists strong coupling between the LO phonon and the free carrier plasmon. By way of fitting parameter simulations, the AlGaN A_1_(LO) line shape in Figure 5, as the LO-phonon–plasmon coupled mode, can be fitted like that in [30], to obtain the carrier concentrations in GaN, AlN, and SiC binary semiconductors. In the present article, we applied this optical method to acquire the electronic carrier densities in ternary AlGaN compounds successfully.

**Figure 6:**
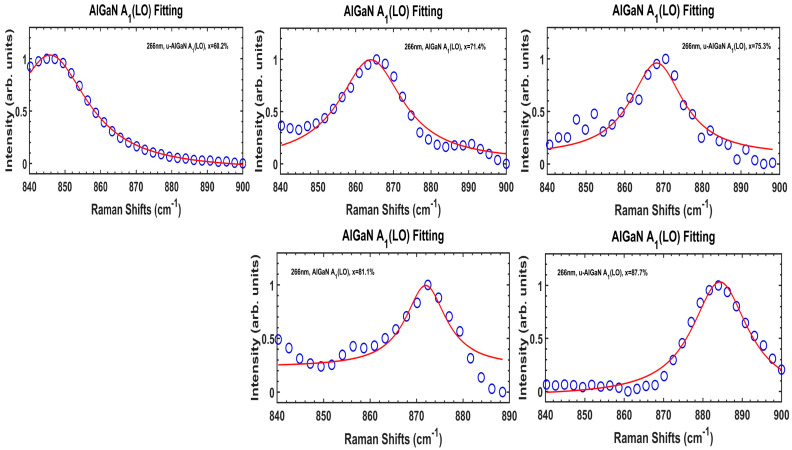
Fitted AlGaN A_1_(LO) modes from DUV 266 nm excitation Raman scattering spectra of five Al_x_Ga_1−x_N/AlN/sapphire samples with high x(Al) between 60.2% and 87.7%, respectively. Fitted values and calculated results of plasmon frequency/damping constant, phonon lifetime, and carrier concentration are listed in Table 3.

Figure 6 shows fitted AlGaN A_1_(LO) modes from Raman scattering data (ex. 266 nm) of five Al_x_Ga_1−x_N/AlN/sapphire samples with high x(Al) between 60.2% and 87.7%, respectively, by using the above Formulas (7)–(11).

**Table 3:** Values of Al_x_Ga_1−x_N A_1_(LO) peak/FWHM, and calculated results of plasmon frequency/damping constant, phonon lifetime, and carrier concentration of five AlGaN films with high x(Al) between 60.2 and 87.7%.

Sample Name (x%)	A60 (60.2%)	A71 (71.4%)	A75 (75.3%)	A81 (81.1%)	A87 (87.7%)
A_1_(LO) peak (cm^−1^)	845.77	864.05	868.25	871.98	884.32
A_1_(LO) FWHM (cm^−1^)	25.88	20.82	16.67	10.64	17.26
ω_p_ (THz)	0.159	0.163	0.164	0.164	0.167
γ_p_ (THz)	4.88	3.92	3.14	2.01	3.25
τ_phonon_ (ps)	0.205	0.255	0.318	0.498	3.07
Fitting Accuracy	97.01%	87.24%	79.92%	77.21%	87.92%
*N* (×10^18^ cm^−3^)	7.51	9.17	10.9	12.2	15.5

Note: ω_p_ (plasmon frequency), γ_p_ (plasmon damping constant), τ_phonon_ (phonon lifetime), *N* (carrier concentration).

**Figure 7:**
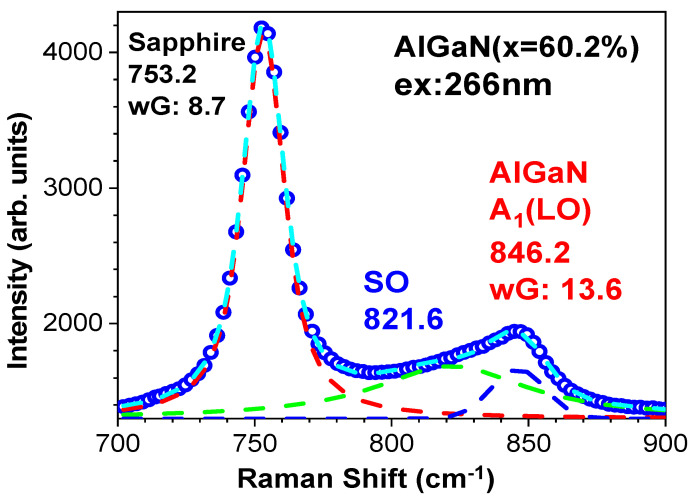
Raman spectral data at 700–900 cm^−1^ from the A60 (x = 60.2%) sample under the 266 nm excitation, and Voigt fittings of three modes of sapphire at 753 cm^−1^, AlGaN A1(LO) at 846 cm^−1^, and the surface optical (SO) mode between them (at 822 cm^−1^).

It is shown in Figure 5 that the A60 (x = 60.2%) sample has its AlGaN A1(LO) mode between 800 and 80 cm^−1^ with heavy asymmetric line shape. Indeed, this is indicative of an additional mode involved in the left wing of the AlGaN A1(LO) peak. A. K. Sivadasan et al. [4] demonstrated the surface optical phonon modes in hexagonal-shaped Al_0.97_Ga_0.03_N nanostructures, located below the AlGaN A1(LO) peak. Figure 7 presents Raman spectral data at 700–900 cm^−1^ from the A60 (x = 60.2%) sample under the 266 nm excitation, and Voigt fittings of three modes including sapphire at 753 cm^−1^, AlGaN A1(LO) at 846 cm^−1^, and the surface optical (SO) mode between them (at 822 cm^−1^). Because of the influence of the SO mode, our above calculations on the plasmon frequency and carrier concentration from the A60 sample could be deviated. Therefore, we repeat the fitting on the A60’s AlGaN A1(LO) mode separated from Voigt fits in Figure 7 to add into Figure 6.

In Figure 5, between 600 cm^−1^ and 700 cm^−1^, i.e., between two strong modes at ~578 cm^−1^ and ~750 cm^−1^ from the sapphire substrate [30,33], there are two modes observed. These are the AlN E_2_(high) mode located at ~650 cm^−1^ and an AlGaN E_2_(high) mode with the peak frequency varied on x(Al) from ~600 cm^−1^ to ~630 cm^−1^ as x(Al) increases from 60.2% to 87.7% in five Al_x_Ga_1−x_N. To investigate these two E_2_(high) modes in depth and clearly, we perform Voigt mode fittings on them.

**Figure 8:**
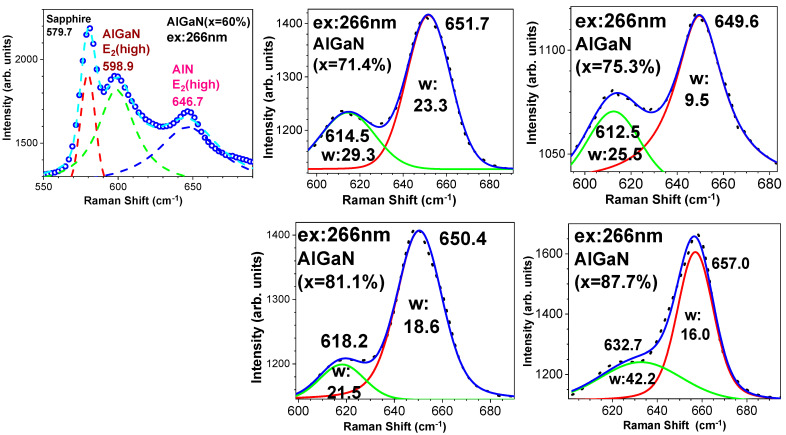
Experimental Raman (ex. 266 nm) data and Voigt mode fittings for AlGaN and AlN E_2_(high) modes in five Al_x_Ga_1−x_N/AlN/sapphire samples with x(Al) of 60.2%, 71.4%, 75.3%, 81.1%, and 87.7%, respectively.

Figure 8 displays these Voigt contours for five Al_x_Ga_1−x_N/AlN hetero-structural samples on sapphire substrates. The A60 (x = 60.2%) sample, due to the big influence of the sapphire 580 cm^−1^ mode, is fitted with three Voigt modes, while the other four samples are all fitted with two Voigt mode contours. It is found that three samples with x(Al) of 71.4%, 75.3%, and 81.1% have the AlN E_2_(high) mode located at 650 ± 1 cm^−1^ only, while the A60 (x = 60.2%) sample has its AlN E_2_(high) mode at ~3 cm^−1^ lower than the standard AlN E_2_(high) of 650 cm^−1^, and the A87 (x = 87.7%) sample has its AlN E_2_(high) at ~7 cm^−1^ higher than the standard AlN E_2_(high) of 650 cm^−1^. Also, the AlGaN E_2_(high) mode has its peak frequency varied at 599–615–613–618–633 cm^−1^ as x(Al) increases from 60.2% to 87.7% in five Al_x_Ga_1−x_N. These phenomena might be caused by the differences in layer axial stresses and lattice constants in Al_x_Ga_1−x_N with different x(Al) amounts. This reveals that in the sample A60, the AlN buffer layer has a tensile stress, because of its AlN E_2_(high) with ~3 cm^−1^ lower than the standard AlN E_2_(high) value, which might be due to its larger lattice difference with the top Al_0.6_Ga_0.4_N thicker layer, while in the sample A87, the AlN buffer layer has a compressive stress, indicated by its AlN E_2_(high) that is ~7 cm^−1^ higher than the standard AlN E_2_(high) value.

Our five Al_x_Ga_1−x_N/AlN/sapphire samples possess AlN buffer layers (mixed with the AlN nucleation layer). By way of the spatial correlation model (SCM) analyses on AlN E_2_(high) modes, the Raman spectral intensity, characteristics of AlN layer quality, can be presented as

(12) Iω∝∫01exp−q2L24d3qω−ωq2+Γ0/22

where *q* is in units of 2π/a, a is the lattice constant, *L* is the correlation length, indicating the phonon propagation length which characterizes the material crystalline perfection, and Г_0_ is the damping constant. The dispersion relation for optical phonons has an analytical form:

ω^2^(q) = A + {A^2^ − B [1 − con(πq)]}^1/2^, (13)

or ω(q) = A − Bq^2^ (14)

where A and B are adjustable parameters [30]. This spatial correlation model (SCM) was employed by us to investigate some semiconductors and oxides, including InGaN [36], SiC [37], InAlN [38], GaN-AlN superlattices [39], GaN/GaAs [40], and so on [30]. From Figure 5 and Figure 8, it is obvious that the AlGaN E_2_(high) and AlN E_2_(high) modes are overlapped partially for all samples and that for the A60 (x(Al) = 60.2%) sample, the AlGaN E_2_(high) mode is overlapped with both the sapphire 580 cm^−1^ mode in the left wing and the AlN E_2_(high) mode in the right wing. Therefore, we conduct the SCM fits on each separated E_2_(high) mode fitted from Voigt contours in Figure 8.

**Figure 9:**
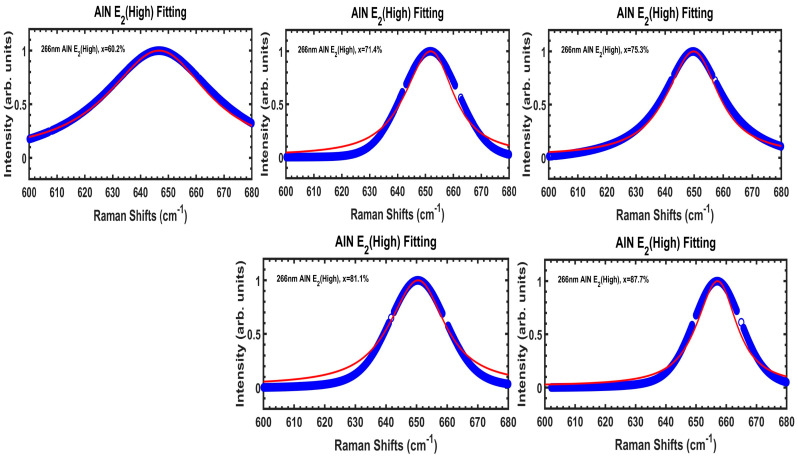
DUV 266 nm excitation Raman spectral information of AlN E_2_(high) modes, with experimental data (fitted from Voigt contours at Figure 8) in blue symbols and SCM fits by red lines, for our five Al_x_Ga_1−x_N/AlN/sapphire samples. The calculated parameters based upon SCM are listed in Table 2.

Figure 9 exhibits DUV 266 nm excitation Raman spectral information of AlN E_2_(high) modes, with experimental data (fitted from Voigt contours at Figure 8) in blue symbols and SCM fits by red lines, for our five Al_x_Ga_1−x_N/AlN/sapphire samples. The calculated parameters based upon SCM are listed in Table 4. It is found that the correlation length L values are increasing gradually as x(Al) increases from 60.2% to 87.7%.

**Figure 10:**
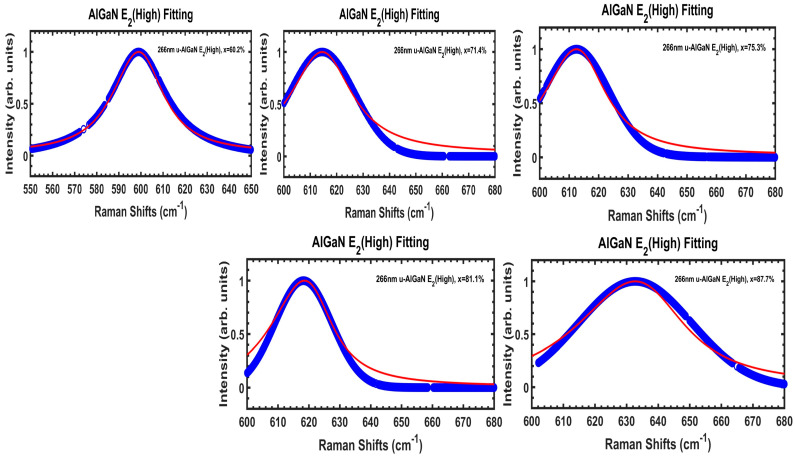
DUV 266 nm excitation Raman spectral information of AlGaN E_2_(high) modes, with experimental data (fitted from Voigt contours at Figure 8) in blue symbols and SCM fits by red lines, for five Al_x_Ga_1−x_N/AlN/sapphire samples. The calculated parameters based upon SCM are listed in Table 5.

Figure 10 exhibits DUV 266 nm excitation Raman spectral information of AlGaN E_2_(high) modes, with experimental data (fitted from Voigt contours at Figure 8) in blue symbols and SCM fits by red lines, for five Al_x_Ga_1−x_N/AlN/sapphire samples. Because the A60 (x = 60.2%) sample has its AlGaN E_2_(high) mode mixed with the sapphire 578 cm^−1^ mode, its AlGaN E_2_(high) mode spectrum is from Voigt fitted contours in Figure 8. The calculated parameters based upon SCM are listed in Table 5. It is found that both the correlation length L and damping constant Г_0_ values are gradually increased with x(Al) = 60.2% to 87.7% for these five Al_x_Ga_1−x_N/AlN samples.

### 3.4. Photoluminescence Analysis

**Figure 11:**
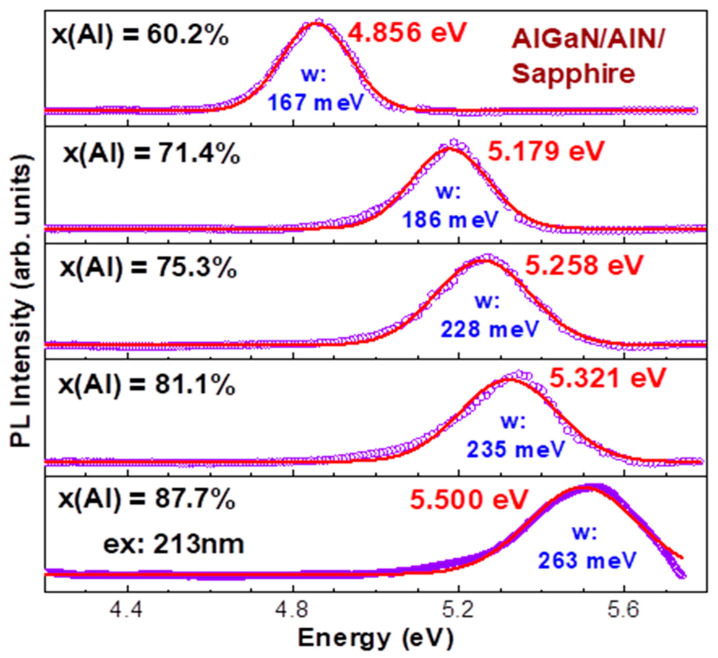
RT photoluminescence (PL) spectra under 213 nm excitation for five Al_x_Ga_1−x_N/AlN/sapphire samples with x(Al) between 60.2 and 87.7%, respectively.

**Figure 12:**
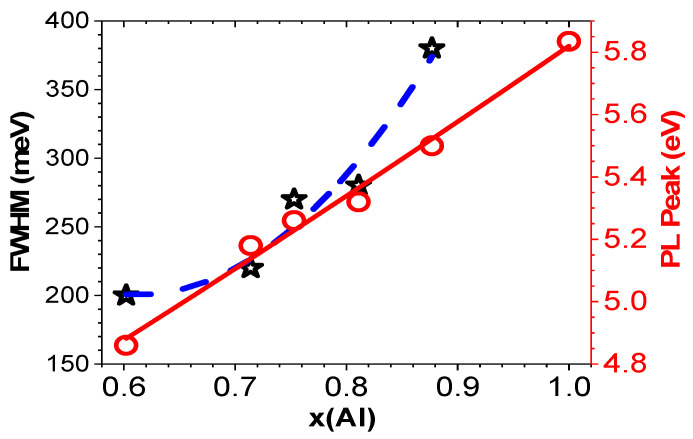
Relationships of the PL peak energy and full width at half maximum (FWHM, i.e., w in Figure 11) values vs. x(Al) for Al_x_Ga_1−x_N/AlN/sapphire, in which an E peak point of x = 1.0 is from previously measured AlN/sapphire under 193 nm excitation [29]. Red open circles are for PL peaks and black stars are for FWHM. Red line and blue dashed line are guides for eye.

Because of tough limitations and difficulties in the experimental setup, there appear big challenges in the literature for DUV PL measurements beyond 5 eV, or shorter than 248 nm, on Al-rich AlGaN materials [6,8,9,14,41], including cathodoluminescence (CL) [12] and electroluminescence (EL) [17,18,42]. Figure 11 presents the RT photoluminescence (PL) spectra under 213 nm excitation for five Al_x_Ga_1−x_N/AlN/sapphire samples with x(Al) between 60.2 and 87.7%, respectively. All PL peaks are fitted using Gaussians, with all fitted peak energy in eV and full width at half maximum (FWHM), i.e., “w: in meV”, marked inside the figure. These values are also displayed in Figure 12, in which a data point at x(Al) = 1.0 is included from an AlN/sapphire, measured under the excitation of 193 nm and reported by us in 2021 [29].

Figure 12 shows the relationship of PL peak energy (eV) vs. x(Al) as follows:

E_PL_ = 3.606 + 1.97x + 0.24x^2^ (eV) (15)

Also, the dependence of full width at half maximum (FWHM, i.e., w in Figure 11) values vs. x(Al) in Figure 12 obeys a relationship:

W = 1119 − 3006x + 2459x ^2^ (meV) (16)

In addition, we measured RT PL spectra under the excitation of 193 nm for two Al_x_Ga_1−x_N/AlN/sapphire samples, with highest x(Al) values of 81.1% and 87.7%. Compared with Figure 11 under the 213 nm excitation, the PL peak energies are slightly lower, with 25 meV and 51 meV, i.e., only 0.5% and 1%, respectively, within experimental errors. The PL band widths are narrower than 7 meV for the 81.1% sample and wider than 20 meV for the 87.7% sample, i.e., 7–8%. Therefore, these measured data under the 193 nm excitation are not shown here.

### 3.5. Temperature-Dependent Photoluminescence Analysis

**Figure 13:**
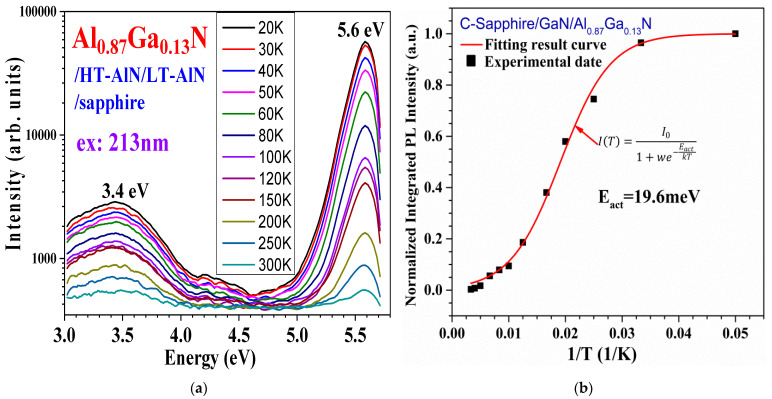
(**a**) Variable temperature (20–300 K) PL spectra of the Al_0.87_Ga_0.13_N/AlN/sapphire sample. (**b**) Normalized integrated PL intensity vs. 1/T, fitted with Arrhenius formulism, obtaining the activation energy of E_act_ = 19.6 meV.

Figure 13a presents temperature-dependent photoluminescence (TDPL) spectra with variable temperature (VT) between 20 and 300 K for the Al_0.87_Ga_0.13_N/AlN/C-sapphire sample. The light-emitting peak at 5.6 eV is band-edge luminescence, and the luminescence peak at 3.4 eV may be defects-related emissions. On the main band, the relationship of normalized integrated PL intensity vs. 1/T is fitted with Arrhenius formulism, obtaining the activation energy of E_act_ = 19.6 meV for this sample, displayed in Figure 13b. Recently, R. Ishii et al. [43] conducted TDPL and time-resolution (TR) PL over 10–500 K for an Al_0.48_Ga_0.52_N MQW on AlN/sapphire, obtained with the radiative process activation energy of 14 meV and nonradiative process 253 meV, respectively.

**Figure 14:**
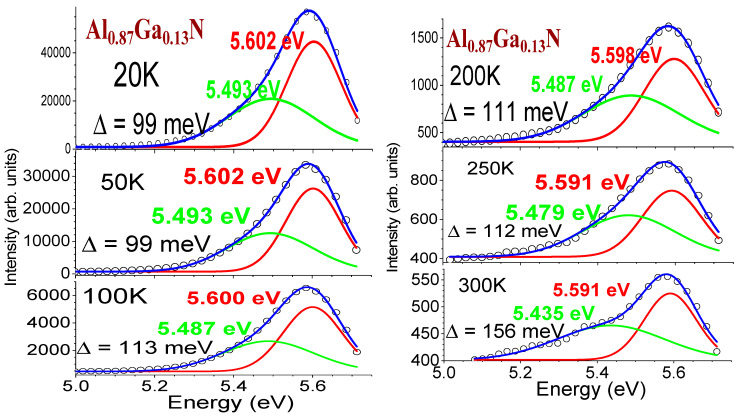
Gaussian fits on the main PL bands at 20, 50, 100, 200, 250, and 300 K, respectively, for the Al_0.87_Ga_0.13_N/AlN/sapphire sample. Each spectrum is deconvoluted to two bands, with the strong one at 5.60–5.59 eV and a weaker one at 5.49–5.48 eV for 20–250 K and at ~5.44 eV for 300 K, respectively.

Furthermore, the main bands near 5.6 eV at Figure 13a seem asymmetrical; based upon these TDPL data (ex. 213 nm), we conducted Gaussian fits on the main PL bands at 20, 50, 100, 200, 250, and 300 K, respectively, for this Al_0.87_Ga_0.13_N/AlN/sapphire sample (displayed in Figure 14). All spectra are deconvoluted to two bands, with the strong one at 5.60–5.59 eV and the weaker one at 5.49–5.48 eV for 20–250 K, respectively. For the spectrum at 300 K, the weak intensity one is seen, with the peak at 5.435 eV.

### 3.6. Time-Resolved Photoluminescence Analysis

**Figure 15:**
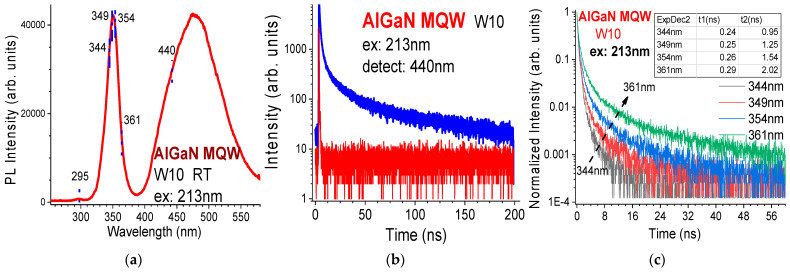
(**a**) RT PL of an AlGaN MQW with MQW emission peak at 350 nm, AlGaN-barrier peak at 295 nm, and a broad band over 400–580 nm. (**b**) RT TRPL decay spectra from the 213 nm pulse laser (red) and the AlGaN MQW sample W10, detected at 440 nm (blue). (**c**) RT TRPL detected at four wavelengths for AlGaN MQW sample W10.

Deep ultraviolet (DUV) time-resolved photoluminescence (TRPL) spectroscopy is a powerful and attractive technology in the investigation of AlN and Al-rich AlGaN materials [44] and high x(Al) AlGaN MQWs [28,43,45,46]. J.W. Lee etc. [45] used the 266 nm pulsed laser excitation to study the PL decays from DUV LED. We established a combined photoluminescence (PL) and time-resolved photoluminescence (TRPL) spectroscopic system with deep ultraviolet (DUV) 213 nm excitation to measure AlGaN multiple quantum wells (MQWs) and related AlGaN epi-films [28,46]. Figure 15a–c show the room temperature (RT) PL and TRPL of an AlGaN MQW, W10. The RT PL of an AlGaN MQW with MQW emission peak at 350 nm, AlGaN-barrier peak at 295 nm, and a broad band over 400–580 nm is exhibited in Figure 15a. Figure 15b displays RT TRPL decay spectra from the 213 nm pulse laser (red) and the AlGaN MQW sample W10, detected at 440 nm (blue). Figure 15c presents RT TRPL decay curves, detected at four wavelengths from the AlGaN MQW sample W10. Each decay spectrum can be fitted with a double-exponential function [28,46]:

I(t) = I_10_exp(−t/τ_1_) + I_20_exp(−t/τ_2_) (17)

with two carrier lifetimes of τ_1_ and τ_2_ obtained, which are listed inside the insert table of Figure 15c. It can be seen that fast decay times of 0.24–0.29 ns (10^−9^ s) and slow decay times of 0.95–2.0 ns are obtained. These values are comparable with those reported previously for other AlGaN MQW samples [43,45].

## 4. Conclusions

In summary, a series of Al_x_Ga_1−x_N films with high x(Al) (60%, 71%, 75%, 81%, 87%) fractions grown on C-plane sapphire substrates with AlN nucleation layer and AlN buffer layer by metal–organic chemical vapor deposition (MOCVD) were prepared and investigated. They were initially characterized by high-resolution X-ray diffraction (HR-XRD) and Raman scattering (RS). We deduced a set of formulas for precisely determining the x(Al) in AlGaN from three orders of HR-XRD data. It was identified that DUV (266 nm) excitation RS clearly exhibits AlGaN Raman features much better than visible RS. Via Voigt fitting, the surface optical (SO) mode in the AlGaN sample with the lowest x(Al) = 60% is revealed. From the simulation on the AlGaN longitudinal optical (LO) phonon modes, the carrier concentrations of AlGaN layers in this set of high x(Al) samples were determined. The Voigt fittings separate the AlGaN and AlN E2(high) modes in overcoming their overlaps. Subsequently, the spatial correlation model (SCM) analyses were applied on the AlGaN and AlN E_2_(high) modes independently and probing two-layer properties. The DUV 213 nm (5.8 eV) laser was employed to study the room temperature (RT) photoluminescence (PL) and temperature-dependent photoluminescence (TDPL) properties of these high x(Al) Al_x_Ga_1−x_N films with large energy gaps E_g_ in the range of 5.0–5.6 eV. The obtained PL bands were deconvoluted with Gaussian bands, indicating the cross-band gap emission and phonon replicas as well as variation with x(Al). TDPL spectra at 20–300 K of an Al_0.87_Ga_0.13_N exhibited the T-dependences of the band-edge luminescence near 5.6 eV and the phonon replicas. According to the Arrhenius fitting diagram of the TDPL spectra, the activation energy (19.6 meV) associated with the luminescent process was acquired. Further, the combined PL and time-resolved photoluminescence (TRPL) spectroscopic system with DUV 213 nm pulse excitation was applied to measure AlGaN multiple quantum wells (MQWs). RT TRPL decay spectra were obtained at four wavelengths and fitted by two exponentials, with fast decay times of 0.24–0.29 ns and slow decay times of 0.95–2.0 ns obtained. Comprehensive studies on the crystalline and optical properties of Al-rich AlGaN epi-films and a typical AlGaN MQW were achieved with unique and significant results, which provides useful references to growers and investigators in the III-nitrides and other materials fields.

## Figures and Tables

**Table 1:** Values of Al_x_Ga_1−x_N (0002) 2θ peak/FWHM, and calculated results of screw dislocation density of four AlGaN films with x(Al) of 35.0%, 71.4%, 81.1%, and 87.7%.

Sample Name (x%)	A35 (35.0%)	A71 (71.4%)	A81 (81.1%)	A87 (87.7%)
AlGaN Peak 2θ (0002) (°)	35.077	35.749	35.749	35.884
AlGaN FWHM 2θ (0002) (°)	0.126	0.129	0.129	0.137
AlGaN β: (2θ_FWHM_*π/180, Rad)	0.002198	0.002179	0.002179	0.002913
AlGaN β^2^ (×10^−6^)	4.83	4.75	4.75	8.49
AlGaN *N* (×10^18^ cm^−3^)	4.12	4.05	4.05	7.24

Note: for AlGaN, b = 0.5185 nm = 0.5185 × 10^−7^ cm, b^2^ = 0.2688 × 10^−14^ cm^2^, 4.36b^2^ = 1.172 × 10^−14^ cm^2^.

**Table 2:** Values of AlN (0002) 2θ peak/FWHM, and calculated results of screw dislocation density of three AlGaN films with x(Al) of 71.4%, 81.1%, and 87.7%.

Sample Name (x%)	A71 (71.4%)	A81 (81.1%)	A87 (87.7%)
AlN Peak 2θ (0002) (°)	36.070	36.070	36.094
AlN FWHM 2θ (0002) (°)	0.157	0.157	0.141
AlN β: (2θ_FWHM_*π/180, Rad)	0.002739	0.002739	0.002460
AlN β^2^ (×10^−6^)	7.50	7.50	6.05
AlN *N* (×10^18^ cm^−3^)	6.93	6.93	5.59

Note: for AlN, b = 0.4982 nm = 0.4982 × 10^−7^ cm, b^2^ = 0.2482 × 10^−14^ cm^2^, 4.36b^2^ = 1.082 × 10^−14^ cm^2^.

**Table 4:** AlN E_2_(high) peak, FWHM, and calculated parameters based upon the spatial correlation model (SCM).

Sample Name (x%)	A60 (60.2%)	A71 (71.4%)	A75 (75.3%)	A81 (81.1%)	A87 (87.7%)
A (cm^−1^)	646.6	651.6	649.6	650.5	657.1
B (cm^−1^)	103	107	109	110	111
L (Å)	10	12	13	13.5	15
Г_0_ (cm^−1^)	22	18	19	19.5	20

**Table 5:** AlGaN E_2_(high) peak, FWHM, and calculated parameters based upon the spatial correlation model (SCM).

Sample Name (x%)	A60 (6.2%)	A71 (71.4%)	A75 (75.3%)	A81 (81.1%)	A87 (87.7%)
A (cm^−1^)	599	614.7	614.9	619.2	638.5
B (cm^−1^)	108	108.5	109	110	112
L (Å)	12	13	13.5	14	14.5
Г_0_ (cm^−1^)	25	26	26.5	27	32

## Data Availability

The data presented in this study are available on request from the corresponding author.
